# Vps10-mediated targeting of Pep4 determines the activity of the vacuole in a substrate-dependent manner

**DOI:** 10.1038/s41598-019-47184-7

**Published:** 2019-07-22

**Authors:** Fahd Boutouja, Christian M. Stiehm, Thomas Mastalski, Rebecca Brinkmeier, Christina Reidick, Fouzi El Magraoui, Harald W. Platta

**Affiliations:** 1 Biochemie Intrazellulärer Transportprozesse, Ruhr-Universität Bochum, 44801 Bochum, Germany; 2 Biomedizinische Forschung, Leibniz-Institute for Analytical Sciences (ISAS-e.V.), 44139 Dortmund, Germany

**Keywords:** Pexophagy, Mitophagy, Protein transport, Proteases, Peroxisomes

## Abstract

The vacuole is the hydrolytic compartment of yeast cells and has a similar function as the lysosome of higher eukaryotes in detoxification and recycling of macromolecules. We analysed the contribution of single vacuolar enzymes to pexophagy and identified the phospholipase Atg15, the V-ATPase factor Vma2 and the serine-protease Prb1 along with the already known aspartyl-protease Pep4 (Proteinase A) to be required for this pathway. We also analysed the trafficking receptor Vps10, which is required for an efficient vacuolar targeting of the precursor form of Pep4. Here we demonstrate a novel context-dependent role of Vps10 in autophagy. We show that reduced maturation of Pep4 in a *VPS10*-deletion strain affects the proteolytic activity of the vacuole depending on the type and amount of substrate. The *VPS10*-deletion has no effect on the degradation of the cytosolic protein Pgk1 via bulk autophagy or on the degradation of ribosomes via ribophagy. In contrast, the degradation of an excess of peroxisomes via pexophagy as well as mitochondria via mitophagy was significantly hampered in a *VPS10*-deletion strain and correlated with a decreased maturation level of Pep4. The results show that Vps10-mediated targeting of Pep4 limits the proteolytic capacity of the vacuole in a substrate-dependent manner.

## Introduction

The main function of the yeast vacuole is the hydrolytic breakdown of its cargoes. Therefore, it can be regarded as the functional counterpart of plant vacuoles or the lysosomes of higher eukaryotes. Additional functional tasks of the vacuole include its role in nutrient storage, recycling of macromolecules, protein homeostasis and detoxification reactions^1,2^.

Approximately 200 yeast genes are annotated to encode for proteins with vacuolar localization. This includes several transporters, membrane fusion complexes or organelle-organelle contact sites. Moreover, several lipases, nucleases and proteases are located within the vacuolar lumen^3^.

There are several known pathways for the transport of proteins to the vacuole. One of them is the so called CPY pathway, based on the well characterised substrate carboxypeptidase Y^4,5^. The vacuolar targeting of Cpy1 requires the interaction with the type I transmembrane sorting receptor Vps10 (vacuolar protein sorting 10)^6^. Vps10 is the founding member of Vps10-domain receptors of higher eukaryotes, like *e.g*. Sortilin. Several of these receptors are connected to neurodegenerative, cardiovascular and metabolic diseases in humans^7,8^. Vps10 cycles between the Golgi and the prevacuolar/endosomal compartment. Another important cargo of Vps10 is the aspartyl endopeptidase Pep4, which is also referred to as Proteinase A (PrA)^9^. Pep4 is regarded as “master protease” since it is required for the activation of most of the other vacuolar proteases via limited proteolysis. The proteases, including Pep4, are usually transported in their inactive precursor form as zymogen^10^. The inhibitory peptide is removed via limited proteolysis within the vacuole, thus allowing a compartmentalised activation. Pep4 undergoes auto-activation, followed by direct cleavage and activation the other proteases. Therefore, the deletion of the *PEP4* gene results in a block of the proteolytic capacity of the vacuole. The substrates, which mainly consist of endocytosed cargo brought via multivesicular bodies or intracellular cargo brought via autophagosomes^2^, are not degraded anymore.

The sorting receptor Vps10 is essential for the vacuolar targeting of Cpy1^6,11^. It is not essential for the targeting of Pep4, but it is required for an efficient targeting of Pep4 to the vacuole^9^. Therefore, alternative receptors, like the telomere-linked Vps10-homologues Vth1 and Vth2^9,12^ or, alternatively, the homologue of the mammalian mannose-6-phosphate receptors, Mrl1^13^, have been proposed as potential candidates with a partially redundant function in the transport of Pep4.

This might be the reason, why it is known that Pep4 is essential for all tested autophagy pathways, while Vps10 has not been linked to this function yet. Here we demonstrate for the first time that Vps10 does play a role in the vacuolar degradation of autophagic cargoes in a context-dependent manner. We show that non-selective macroautophagy (bulk autophagy) and ribophagy are Vps10-independent, while the degradation of an excess of peroxisomes via pexophagy and mitochondria via mitophagy is correlating with the efficient maturation of Pep4 in a Vps10-dependent manner.

## Results

### Analysis of the contribution of single vacuolar enzymes to the autophagic degradation of peroxisomes

The vacuole is a hydrolytic compartment, in which a variety of endocytotic and autophagic cargoes are degraded. We tested the individual contribution of central vacuolar enzymes by using the pathway for the selective autophagic degradation of peroxisomes (pexophagy) as test system in *Saccharomyces cerevisiae*.

**Figure 1:**
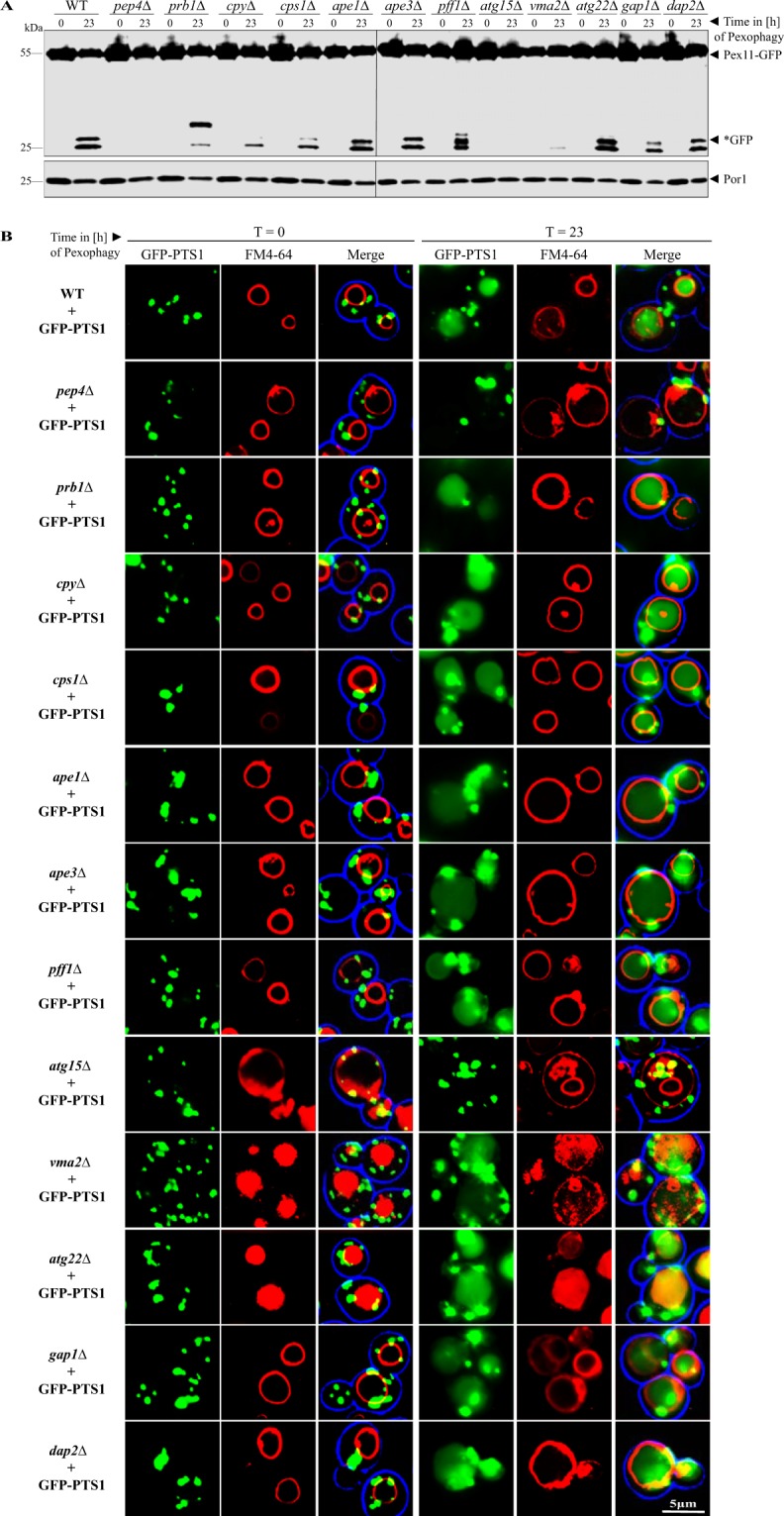
Analysis of the influence of vacuolar enzymes on the selective autophagic degradation of peroxisomes. (**A**) The possible contribution of the indicated strains deleted for vacuolar enzymes on pexophagy was monitored via the stability of the peroxisomal membrane protein Pex11, which was genetically fused to GFP. After proliferation of peroxisomes, cells were shifted to pexophagy-medium. In wild-type cells (WT), the Pex11-part of the fusion protein is degraded in the vacuole together with the rest of the organelle, while the GFP-portion is largely stable (*GFP). This process is blocked in *pep4*Δ cells. In addition, we find that Atg15, Prb1 and Vma2 are required to complete pexophagy. (**B**) Peroxisomes were labeled with the matrix protein GFP-PTS1, which is the standard marker for fluorescence microscopy. The vacuolar membrane was stained with FM4-64. Prior to the induction of pexophagy (t = 0 h), the peroxisomes are visible as green cytosolic dots and the vacuolar lumen does not display any signals. At the end of the pexophagy assay (t = 23 h), some peroxisomal signals remain in the cytosol, while the vacuole of WT cells is filled with a diffuse green staining, indicating peroxisomal breakdown. The vacuole is still empty in *pep4*Δ, *atg15*Δ and nearly empty in *vma2*Δ cells, indicating a pronounced defect in pexophagy. The full-length blots are presented in Supplementary Fig. S2. The combined parts in Fig. 1A stem from the same experiment and the corresponding samples were analyzed in parallel.

The pexophagy assay (Fig. 1A) utilizes the peroxisomal membrane protein Pex11 genetically fused to GFP (green fluorescent protein) as marker. First, the proliferation of peroxisomes was induced by the shift of glucose-grown cells to glucose-lacking and oleate-containing medium. Peroxisomes proliferate in oleate-medium, which results in a higher number of these organelles^14^. Then, peroxisome degradation was induced by a shift of the cells to an oleate-lacking and glucose-containing medium. Moreover, this pexophagy-medium has reduced nitrogen sources, which is a starvation condition. Peroxisomes are not essential anymore and therefore the excess of these organelles is a substrate for pexophagy^15^. Pex11 is hydrolyzed together with the entire peroxisome within the vacuole of WT cells. Interestingly, the GFP-fusion-tag is not degraded within the vacuole. Therefore, the occurrence of free *GFP can be regarded as a marker for functional pexophagy^16,17^.

We find that the vacuolar activity required for the generation of *GFP via pexophagy has a WT-like level, when the genes for the proteases Ape1, Ape3, Pff1, Dap2 or the amino acid transporters Gap2 and Atg22 are deleted, while the deletion of the proteases Cpy1 (Prc1) and Cps resulted in slightly less *GFP (Fig. 1A). The finding that these factors are not essential for pexophagy is further supported by fluorescence microscopy (Fig. 1B). Peroxisomes are marked with GFP-PTS1 and can be detected as punctate pattern. At the end of the pexophagy assay, the FM4-64-stained vacuoles are filled with a diffuse green signal, showing that parts of the peroxisome population have been broken down within the vacuolar lumen.

We used the *pep4*Δ strain as a negative control. The aspartyl-protease Pep4 (PrA) is known to be required for pexophagy^18^ and therefore we find no diffuse green staining in the vacuolar lumen under the microscope and no *GFP in the sample of the biochemical assay. We discover that the formation of *GFP is nearly abolished in *vma2*Δ cells, which lack the B subunit of the V1 peripheral membrane domain of the vacuolar H^+^-ATPase. Vma2 controls the H^+^ influx and therefore acidification of the vacuolar lumen, which is required for the activity of several hydrolytic enzymes^3^. Therefore, we demonstrate that a proper acidification of the vacuole is a prerequisite for the hydrolytic activity required for the breakdown of peroxisomes. The fluorescence microscopic pictures show FM4-64-stained membrane structures within the vacuolar lumen, while the GFP-signal is nearly absent. Moreover, we identify the phospholipase Atg15 to be essential for pexophagy, as no *GFP is generated in these cells. Atg15 is known to breakdown intravacuolar vesicles^19^ and therefore could be required here for the opening of the pexophagosomal membrane as well as of the peroxisomal membrane itself. In line with this, we find the accumulation of vesicular structures in the microscopic pictures of the *atg15*Δ strain (T = 23 h). As additional observation, we find that the starting points (T = 0 h) of *atg15*Δ as well as of *vma2*Δ and *atg22*Δ cells already seem to show intravacuolar FM4-64 staining. This could be explained by the findings that *vma2*Δ cells do not provide the optimal pH value for hydrolytic enzymes required for the breakdown of intravacuolar vesicles^3^, while *atg22*Δ cells are reported to show an at least delayed breakdown of intravacuolar vesicles^20,21^. Cells lacking the serine-protease Prb1 display green signal within the vacuole in the fluorescence microscopic pictures. However, the biochemical analysis reveals that degradation of the marker Pex11-GFP is reduced. The main degradation product runs at a slightly higher molecular weight than usually observed, indicating an incomplete proteolysis of the fusion protein. This could be a direct effect due to the loss of the Prb1 proteolytic activity or an indirect effect due to the circumstance that Prb1 is known to be involved in the maturation of Pep4^22^. In summary, in addition to Pep4 we find Prb1, Atg15 and Vma2 to be involved in pexophagy.

### Analysis of the involvement of vacuolar hydrolases in the maturation and localization of Pep4: role of the Pep4-receptor Vps10 in vacuolar targeting

**Figure 2:**
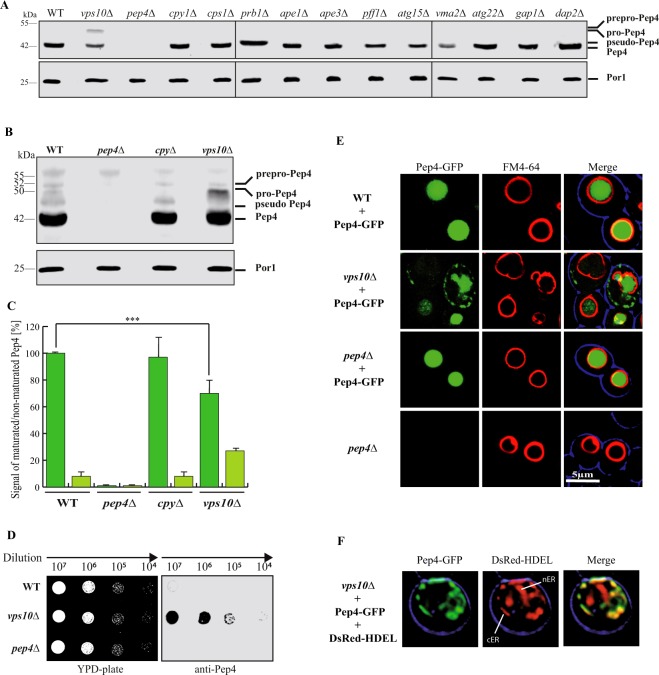
The deletion of *VPS10* results in a reduced vacuolar targeting and maturation of Pep4. (**A**) The different Pep4 (Proteinase **A**) versions were detected in the TCA-precipitated lysates of the indicated strains. Most strains contained fully maturated Pep4 like in wild-type cells. The *prb1*Δ strain contains pseudo-Pep4. The *vps10*Δ strain contains an elevated amount of pre-Pep4. (**B**) In order to prepare for densitometric analysis, SDS gels were loaded with a higher amount of material from samples derived from the indicated strains. (**C**) The statistical analysis of the densitometry data shows that mature Pep4 species are significantly reduced the *vps10*Δ strain when compared with WT. (**D**) Series of dilutions from different strains were spotted on YPD plates with or without nitrocellulose membrane. Finally, the nitrocellulose was incubated with Pep4-antibodies. In contrast to wild-type, secreted Pep4-species can be detected on *vps10*Δ cells. (**E**) The subcellular distribution of Pep4 was analyzed via fluorescence microscopy using Pep4-GFP. The vacuolar membrane was stained red with FM4-64. Pep4-GFP was exclusively found within the vacuole in wild-type cells, while it was also detected in the cytosolic area and additional structures in the *vps10*Δ strain. (**F**) The cortical endoplasmic reticulum (cER) and the nuclear endoplasmic reticulum (nER) were marked with DsRed-HDEL. The non-vacuolar Pep4-GFP population localizes to the cER. The full-length blots of Fig. 2A are presented in Supplementary Fig. S2, while the full-length blots of Fig. 2B are presented in Supplementary Fig. S4. The combined parts in Fig. 2A stem from the same experiment and the corresponding samples were analyzed in parallel.

We wanted to know, if the effect of the analyzed mutants on pexophagy can be correlated with the maturation of the master-protease Pep4. We detected the Pep4-species within the TCA-precipitated lysates of the deletion strains (Fig. 2A). In addition to the set of cells affected in vacuolar proteins, we also tested Vps10, which is the main targeting receptor of the zymogen of Pep4 on its way to the vacuole^9^. Our re-investigation of Pep4 maturation, extended with previously untested deletion strains, shows that the only vacuolar protease required for full Pep4 maturation is Prb1. While the other mutant strains display fully maturated Pep4 (42 kDa), the *prb1*Δ cells contain pseudo-Pep4 (43 kDa)^22^, which is the product of the auto-activation of Pep4. Also Vma2 and Atg15 have no effect, which means that their requirement for pexophagy, which we have described, is based on their individual specific function and not correlated to Pep4 maturation. This allows also the conclusion that obviously the vacuolar pH in *vma2*Δ cells either is still sufficient for Pep4 auto-activation or that Pep4 maturates already during the late steps of the transport to the vacuole within its vesicular carrier. Moreover, *atg15*Δ cells enable the contact of Prb1 with the pseudo-Pep4 species, indicating that either both enzymes are already present in the same vesicular carrier or that these vesicular carriers are opened in an Atg15-independent manner.

Next we wanted to focus on Vps10, which is the main trafficking receptor for the vacuolar targeting of Pep4, by analyzing its impact on the maturation of Pep4 in more detail in order to finally correlate these data with the vacuolar activity in different autophagy pathways.

Earlier studies used pulse labeled cells with ^35^S-labeled amino acids to show that the deletion of *VPS10* reduces the amount of pre-Pep4 which can maturate into Pep4^9,12^. In our study, we focus on the steady state level of Pep4 and find that also under these conditions a significant amount of pre-Pep4 can be detected in the lysate of a *vps10*Δ strain (Fig. 2A), while mainly fully maturated Pep4 can be detected in wild-type (WT) cells or the *cpy1*Δ strain, which is deleted in another Vps10 cargo protein. We then focused on the *vps10*Δ strain and corresponding controls and therefore loaded a doubled amount of material on SDS gels in order to analyze the precursor bands in greater detail with the densitometry of the immunoblot signals (Fig. 2B). We performed a statistical analysis of the densitometry data and could show that the intracellular mature Pep4 species are reduced to 75%, while the intracellular pre-Pep4 species are elevated to 25% (Fig. 2C). A reduced or deficient vacuolar targeting of vacuolar proteins often results in the secretion of the corresponding proteins^9^. We spotted series of dilutions of cells from different strains either directly on YPD-plates or on a nitrocellulose membrane placed on a YPD-plate. After incubation, the nitrocellulose was probed with Pep4-antibodies (Fig. 2D). In contrast to wild-type cells, secreted Pep4-species can be detected in the *vps10*Δ strain. This result shows that the reduced maturation rate of Pep4 is linked to the finding that Pep4-species are secreted in a *VPS10*-deficient strain, as expected. However, we additionally focused on the subcellular localization of the non-secreted Pep4 and analyzed the subcellular distribution of Pep4 species via the fluorescence microscope using a GFP-fused variant of Pep4 (Fig. 2E). Under these conditions, Pep4-GFP can be exclusively found within the vacuole in WT cells. The vacuolar membrane was stained red with FM4-64. In contrast, Pep4-GFP can be detected also in defined structures outside of the vacuole in the *vps10*Δ strain. We marked the endoplasmic reticulum with DsRed-HDEL and found that this Pep4-GFP population co-localized with the cortical endoplasmic reticulum (cER) in *vps10*Δ cells (Fig. 2F)

Therefore, we wanted to analyze if the deletion of the targeting receptor Vps10 and the resulting reduced amount of maturated vacuolar Pep4 have an impact on the proteolytic activity of the vacuole in autophagic pathways.

### Vps10 is required for the efficient degradation of peroxisomes during pexophagy but not for the degradation of the cytosolic protein Pgk1 during bulk autophagy

**Figure 3:**
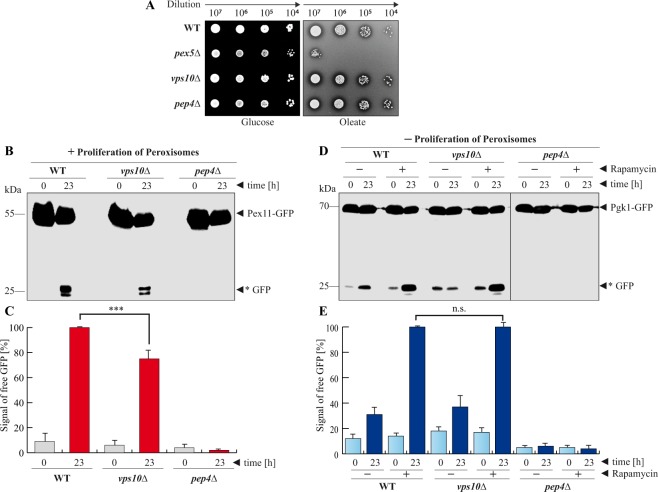
Vps10 is required for efficient pexophagy but not for bulk autophagy. (**A**) Series of dilutions from different strains were spotted on glucose and oleate plates. The results show neither the deletion of *VPS10* or *PEP4* have an influence on the biogenesis of peroxisomes. (**B**) The influence of Vps10 on pexophagy was monitored via the stability of the peroxisomal membrane protein Pex11, which was genetically fused to GFP. After proliferation of peroxisomes (+Proliferation of Peroxisomes), cells were shifted to pexophagy-medium. In wild-type cells (WT), the Pex11-part of the fusion protein is degraded in the vacuole, while the GFP-portion is largely stable (*GFP). This process is blocked in *pep4*Δ cells. The amount of free *GFP is reduced in *vps10*Δ cells. (**C**) The *GFP signals were measured via densitometry. The statistical analysis of the data demonstrates that the amount of generated *GFP is significantly reduced in the *vps10*Δ strain. (**D**) The cytosolic protein Pgk1-GFP shows a certain constitutive turnover. The autophagic degradation of Pgk1-GFP via bulk autophagy was enhanced by addition of rapamycin. While the Pgk1-portion of the fusion protein is degraded in the vacuole, the GFP-portion is stable (*GFP). At the end of the bulk autophagy-assay (+rapamycin/23 h) no difference in the amount of generated *GFP could be detected between WT and *vps10*Δ cells. This result is supported by statistical analysis of the densitometry data. The amount of *GFP does not differ significantly between WT and *vps10*Δ cells. The data demonstrate that the deletion of *VPS10* affects the degradation of peroxisomes via pexophagy and not the degradation of the cytosolic protein Pgk1 via bulk autophagy. The full-length blot of. Figure 3B is presented in Supplementary Fig. S2, while the corresponding full-length blots of Fig. 3D are shown in Supplementary Figure S3. The combined parts in Fig. 3D stem from the same experiment and the corresponding samples were analyzed in parallel.

In order to exclude the possibility that Vps10 has a role in the formation of peroxisomes, we performed a biogenesis test (Fig. 3A). Peroxisomes are the sole site for thebeta-oxidation of fatty acids in yeast. Therefore, peroxisomes are essential for growth when oleate is provided as single carbon source^14^. The deletion of the main import receptor for peroxisomal enzymes, Pex5, results in a complete growth defect on oleate media (Fig. 3A)^23^. The *vps10*Δ strain as well as the *pep4*Δ strain grew similar to the wild-type, which shows that Vps10 and Pep4 are not required for peroxisomal biogenesis and therefore their deletion does not impair the metabolic activity of peroxisomes. Therefore, all possibly observed effects during the pexophagy assays should be specific for the degradation of peroxisomes.

We addressed the question, if the reduced maturation rate of Pep4 in a *vps10*Δ strain has an impact on the selective autophagic degradation of peroxisomes via pexophagy (Fig. 3B). We find that the lysates from the *vps10*Δ strain contain free *GFP. Interestingly, the amount of free *GFP is reduced when compared to the wild-type cells. The densitometric analysis of the *GFP-signals revealed that only 75% of the amount of free *GFP found in wild-type cells can be detected in the *vps10*Δ strain (Fig. 3C). This result suggests that Vps10 is not essential but required for efficient pexophagy.

Next we tested, if the degradation of the cytosolic protein Pgk1(3-phosphoglycerate kinase 1)-GFP via bulk autophagy is affected in a *vps10*Δ strain. Based on the same principle, we used Pgk1-GFP and monitored the occurrence of free *GFP (Fig. 3D). We find that Pgk1-GFP displays a constitutive turnover, which is significantly enhanced when the macrolide rapamycin is used to induce macroautophagy. The so-called non-selective macroautophagy is a bulk degradation pathway of cytosolic components. The results show that the enhanced proteolysis of the Pgk1 part and liberation of the GFP part of the fusion protein depends in presence of rapamycin. No free *GFP was generated in the *pep4*Δ strain. The amount of free *GFP in the *vps10*Δ strain was not significantly different from the level in WT cells (Fig. 3E). This result indicates that the absence of Vps10 has no impact on the efficiency of the Pgk1 breakdown via bulk autophagy.

In summary, the presented results can correlate the less efficient vacuolar targeting and maturation of Pep4 with a hampered degradation of peroxisomes via pexophagy in a *vps10*Δ strain. In contrast, this correlation cannot be found in the context of the degradation of the cytosolic protein Pgk1 via bulk autophagy.

### The Vps10-dependent proteolytic activity of the vacuole depends on the amount of specific substrates

At this point, the finding that the deletion of *VPS10* affects the degradation of peroxisomes but not the degradation of Pgk1 can still be interpreted in two ways. The first possibility is that the hydrolytic efficiency of the vacuoles in *vps10*Δ cells differs depending on the nature of the substrate. It might be more demanding and complex to degrade entire organelles than single soluble proteins. The second possibility is that the conditions of the test assays might have more general and indirect effects. In order to rule out a possible indirect influence we swapped marker proteins and inducing conditions and tested the stability of Pgk1-GFP after shift to pexophagy-medium as well as the stability of Pex11-GFP after addition of rapamycin.

**Figure 4:**
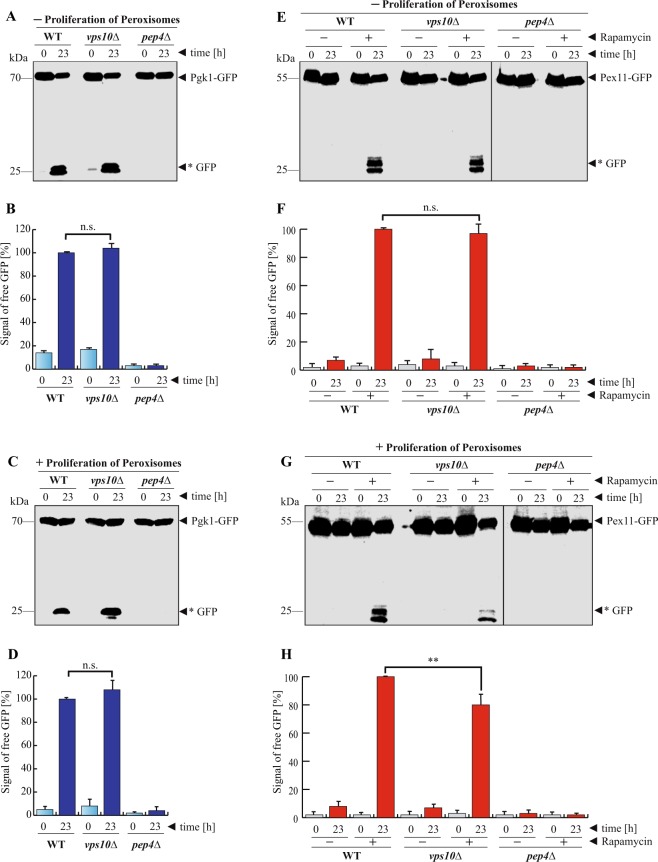
The Vps10-dependent proteolytic capacity of the vacuole depends on the type and amount of substrate. (**A**) The breakdown of the cytosolic Pgk1-GFP was monitored after induction of pexophagy. A portion of Pgk1-GFP was expected to be degraded because of the N-starvation during the pexophagy assay. The data show an equal amount of generated free *GFP after 23 h in WT and *vps10*Δ cells. (**B**) The statistical analysis of the densitometry data shows that the amount of *GFP does not differ significantly between WT and *vps10*Δ cells. (**C**) The breakdown of Pgk1-GFP was monitored under pexophagy-conditions after proliferation of peroxisomes with oleate. The results demonstrate that the autophagic degradation of Pgk1-GFP under these conditions does not differ between WT and *vps10*Δ cells. (**D**) The statistical analysis shows that there is no significant difference between WT and *vps10*Δ cells. (**E**) The breakdown of peroxisomes marked with Pex11-GFP was monitored under general bulk autophagy-conditions, under which cytosolic factors are degraded by the vacuole. Under standard bulk autophagy conditions, no difference between WT and *vps10*Δ cells was detected. (**F**) The statistical analysis of the densitometric data of the Pex11-GFP-derived *GFP shows no significant difference between WT and *vps10*Δ cells. (**G**) When the bulk autophagy-assay with Pex11-GFP-marked peroxisomes was performed after peroxisome proliferation, we could observe a difference. The higher amount of peroxisomes was degraded efficiently in WT cells, while the peroxisomal degradation and generation of *GFP was reduced in *vps10*Δ cells. (**H**) The statistical analysis of the densitometric data of the Pex11-GFP-derived *GFP shows that the amount of generated *GFP is significantly reduced in *vps10*Δ cells. The full-length blots are presented in Supplementary Fig. S3. The combined parts in Fig. 4E and G stem from the same experiment and the corresponding samples were analyzed in parallel.

First, we analyzed the stability of Pgk1-GFP. The cells were grown in glucose-medium and then were shifted to pexophagy-medium (Fig. 4A). Because of the reduced amount of nitrogen-sources in the pexophagy-medium, a certain portion of cytosolic content should be degraded along with the peroxisomes. We find that the proteolysis of the Pgk1-GFP fusion protein does not differ between WT cells and *vps10*Δ cells. Also the statistical analysis of the densitometry data of the *GFP signals shows that there is no significant difference between the generated *GFP in WT and *vps10*Δ cells (Fig. 4B). Because the classical pexophagy-assay for peroxisomes is preceded by the oleate-induced proliferation of peroxisomes, we also tested if this condition affects Pgk1-GFP proteolysis (Fig. 4C). Glucose-grown cells were first transferred to oleate-medium and then to pexophagy-medium. The amount of *GFP in *vps10*Δ cells was not smaller than in WT cells. The analysis of the densitometry data demonstrates that there is no significant difference between the proteolysis of Pgk1-GFP in the WT strain and the *vps10*Δ strain (Fig. 4D). Therefore, the cytosolic protein Pgk1 can be degraded efficiently in *vps10*Δ cells not just after massive induction of bulk autophagy by rapamycin, but also after the shift to pexophagy-medium with reduced nitrogen sources and independently from the oleate treatment.

Next, we tested the degradation of Pex11-GFP-marked peroxisomes after rapamycin treatment. The induction of bulk autophagy is supposed to target all accessible cytosolic content to the vacuole, which should also include a certain amount of organelles like peroxisomes. Glucose-grown cells were exposed to rapamycin, corresponding to the classical macroautophagy-assay (Fig. 4E). In contrast to the result obtained with the classical pexophagy assay (Fig. 3B,C), the amount of generated free *GFP did not change between the WT and the *vps10*Δ strain after addition of rapamycin to glucose-grown cells. This was supported by the statistical analysis of the *GFP densitometry data (Fig. 4F), which did not find a significant difference between the two strains. Peroxisomes are less abundant when grown solely on standard glucose medium than in oleate-containing medium^14^.

We have analyzed the induction of peroxisomal proliferation in the context of our experimental setup (Suppl. Fig. S1). We find that the shift to oleate medium results in a detectable increase of the protein level of the plasmid-encoded Pex11-GFP (Suppl. Fig. S1A) as well as in a strong increase of the protein level of the endogenous peroxin Pex5 (Suppl. Fig. S1B). This should correspond to an elevated proliferation of peroxisomes. Independent from non-inducing (glucose) or inducing (oleate) conditions, we find that Pex11-GFP is always specifically localized to mChery-SKL-labelled peroxisomes in WT, *pep4*Δ and *vps10*Δ cells (Suppl. Fig. S1C).

The pexophagy assay (Fig. 3B,C) was performed after peroxisomal proliferaton had been induced. In order to analyze whether the amount of peroxisomes has an impact on the hydrolytic efficiency of the vacuole, we first induced peroxisome proliferation with oleate and then induced macroautophagy by addition of rapamycin (Fig. 4G). The amount of generated *GFP was reduced in the *vps10*Δ strain compared to WT cells. The statistical analysis of the densitometric data demonstrated that significantly less *GFP is detectable in *vps10*Δ cells (Fig. 4H). Therefore, not only the type of substrate but also the amount of the individual substrate has an important impact on the proteolytic capacity of the vacuole in *S. cerevisiae*.

### The presence of Vps10 is not required for ribophagy

Based on our finding that Vps10-mediated targeting of Pep4 affects pexophagy and bulk autophagy differently, we wanted to analyze its influence on the autophagic breakdown of other substrates with different complexities.

**Figure 5:**
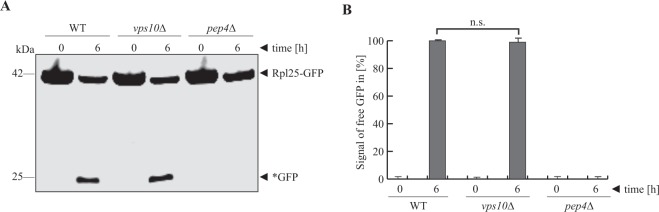
Vps10 is not required for ribophagy. (**A**) The influence of Vps10 on ribophagy was analyzed via the stability of the ribosomal protein Rpl25, which was genetically fused to GFP. In wild-type cells (WT), the Rpl25-part of the fusion protein is degraded in the vacuole, while the GFP-portion is largely stable (*GFP). This process is blocked in *pep4*Δ cells. The amount of free *GFP is the same in *vps10*Δ cells as in WT cells. (**B**) The *GFP signals were measured via densitometry. The statistical analysis of the data demonstrates that the amount of generated *GFP in the *vps10*Δ strain is not significantly different from the amount found in WT cells. The full-length blot is presented in Supplementary Fig. S3.

Mature ribosomes are 3.2 × 10^6^ kDa complexes consisting of ribosomal proteins and rRNAs. They can be selectively degraded via ribophagy^24^. We used the ribosomal protein of the large subunit Rpl25 (ribosomal protein large 25) fused to GFP to directly monitor the ribophagy of the 60 S subunit (Fig. 5A). The amount of the generated free *GFP is comparable in WT and *vps10*Δ cells. This was supported by densitometric analysis (Fig. 5B), which found no significant difference between the two strains. It can be concluded that the vacuoles in *vps10*Δ cells still have enough hydrolytic activity to breakdown ribosomes.

### Vps10 is required for efficient mitophagy

Because we have found that Vps10 is not required for the degradation of single proteins by bulk autophagy or ribosomes by ribophagy, but instead for the breakdown of an excess of peroxisomes via pexophagy, we wanted to test another large and complex substrate, like mitochondria.

**Figure 6:**
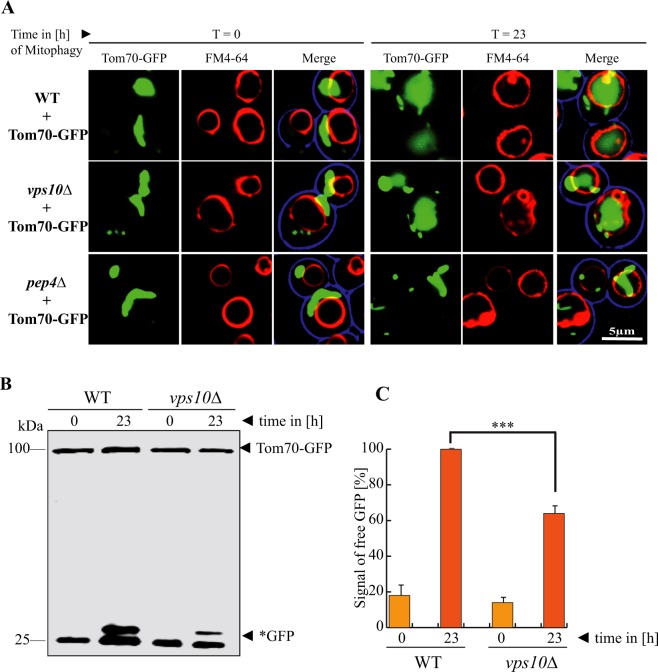
Vps10 is required for efficient mitophagy. (**A**) The fluorescence microscopic analysis of mitophagy was carried out with the mitochondrial protein Tom70, which was genetically fused to GFP. While the diffuse pattern of *GFP indicates a functional mitophagy in the wild-type (WT) strain, the stability of the distinct GFP signals corresponding to intact mitochondria shows a mitophagy defect in *pep4*Δ cells. (**B**) The influence of Vps10 on mitophagy was analyzed via the stability of the mitochondrial fusion protein Tom70-GFP. In WT cells, the Tom70-part of the fusion protein is degraded in the vacuole, while the GFP-portion is largely stable (*GFP). The amount of free *GFP is reduced in *vps10*Δ. (**C**) The *GFP signals were measured via densitometry. The statistical analysis of the data demonstrates that the amount of generated *GFP in the *vps10*Δ strain is significantly reduced when compared with the amount detected in WT cells. The full-length blot is presented in Supplementary Fig. S3.

We utilized the mitochondrial protein Tom70 (translocase of the outer membrane 70) fused to GFP as marker for mitophagy. We monitored mitophagy using the fluorescence microscope (Fig. 6A) and found that mitochondria are degraded in WT cells, as shown by the diffuse green signals within the vacuole, indicating that only free *GFP is still present. In marked contrast, *pep4*Δ cells show only well-defined GFP signals that correspond to intact mitochondria. They are still located in the cytosol or may even be taken up by the vacuole but they are never diffuse and therefore indicate a lack of mitochondrial degradation. The *vps10*Δ cells seem to display a WT-like phenotype, even though the signal intensities within the vacuole seem to be reduced. For a closer inspection, we performed a biochemical analysis, which revealed that less *GFP was generated in the *vps10*Δ strain than in WT cells (Fig. 6B). The amount of free *GFP is significantly reduced when *VPS10* is missing, as demonstrated by the analysis of the densitometric data (Fig. 6C). Therefore, we can conclude that the reduced maturation and vacuolar targeting of Pep4 in the *vps10*Δ strain does not only affect pexophagy of oleate-induced cells but also the selective autophagic degradation of mitochondria via mitohagy.

## Discussion

In this manuscript, we have identified a role of Vps10 in autophagy and characterized the context-dependent impact of a reduced targeting and maturation of Pep4 on four different autophagic pathways. The data presented in this study describe for the first time the functional impact of the sorting receptor Vps10 on pexophagy and mitophagy.

Previous studies did not identify Vps10 in the context of autophagy, probably because alternative receptors can target parts of the Pep4 population in absence of Vps10^9,12^. Moreover, we also find that Vps10 is not required for ribophagy or bulk autophagy, which is the most intensively analyzed autophagy pathway.

**Figure 7:**
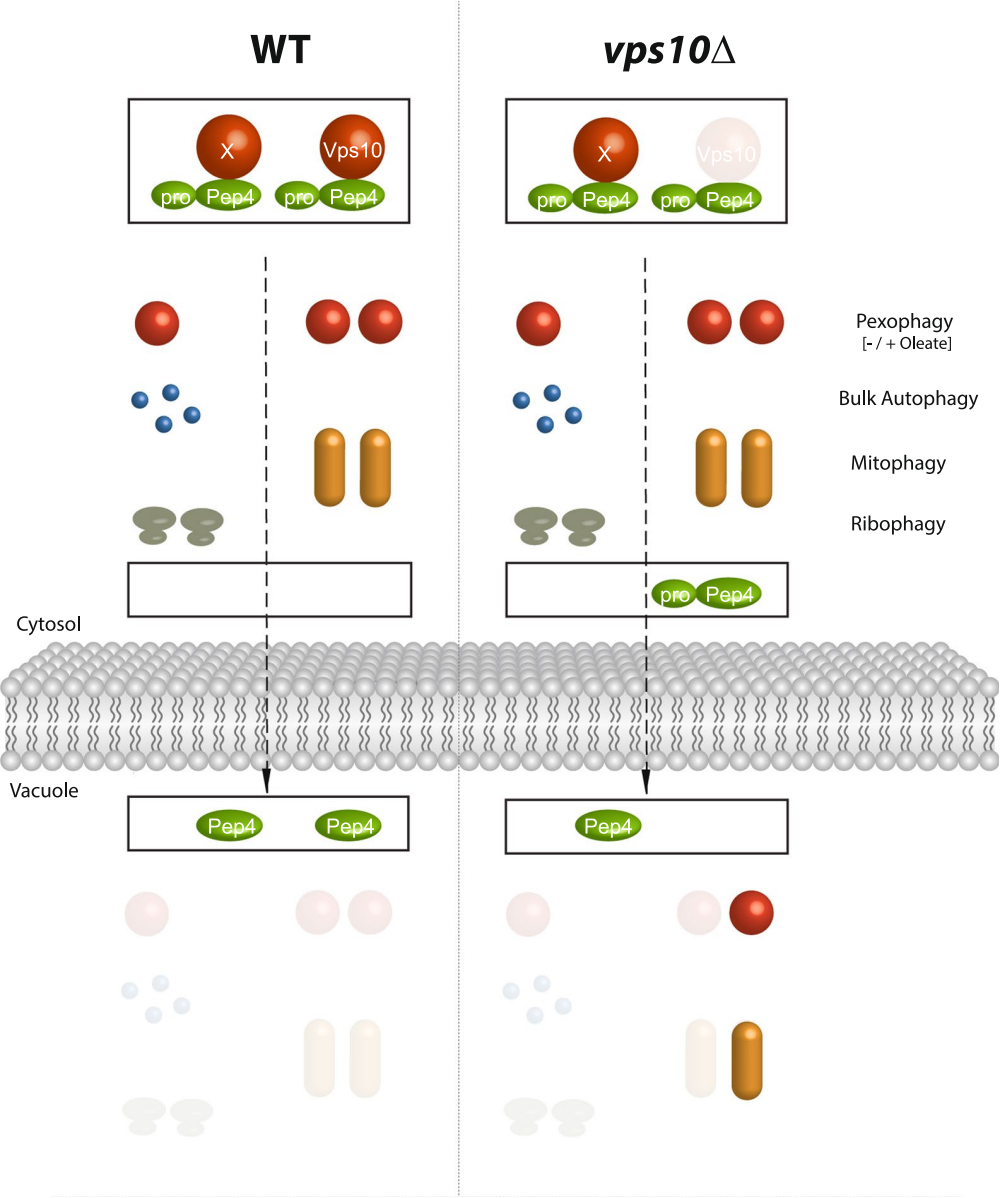
Model: Efficient Vps10-dependent maturation of Pep4 correlates with efficient degradation of an excess of peroxisomes and mitochondria. The vacuolar master protease Pep4 is transported as zymogen (pro-Pep4) from the pre-vacuolar compartment to the vacuolar lumen by Vps10 as well as an unknown, partially redundant receptor (X). We find that the deletion of *VPS10* has no effect on the bulk autophagy of cytosolic proteins, ribophagy or the deletion of a low number of peroxisomes via pexophagy (− oleate). However, after the peroxisome population had increased via the induction of peroxisomal proliferation (+ oleate), the efficiency of the autophagic degradation of peroxisomes via pexophagy was hampered. Moreover, mitophagy took not place efficiently as well. We conclude that the efficiency of Pep4 maturation correlates with classical pexophagy and mitophagy. Therefore, the data indicate that Vps10-depending targeting of Pep4 influences autophagic processes depending on the type and amount of substrate.

We took a special focus on the conditions when the deletion of *VPS10* does or does not affect the autophagic degradation of different cargoes. Our important finding is that the catalytic capacity of the vacuole is depending on the combination of the type and the amount of the substrate in absence of Vps10 (Fig. 7).

We show that the reduction of vacuolar targeting of Pep4 in a *vps10*Δ strain affects the analysed markers for peroxisomal degradation and bulk autophagy in different ways. While low numbers of peroxisomes can be rapidly degraded in *vps10*Δ cells, high amounts of peroxisomes cannot be efficiently degraded in these cells anymore. This is also independent of the induction type (pexophagy-medium or rapamycin treatment). This finding suggests that the reduced amount of vacuolar Pep4 and the enzymes depending on Pep4, can manage few peroxisomes, but not high levels of peroxisomes. Therefore, *vps10*Δ cells do not contain enough hydrolytic activity to cope with a high number of large and complex substrates like peroxisomes. Futhermore, we extend our finding by the result that also mitophagy is affected in the *vps10*Δ strain.

The vacuolar degradation of Pgk1, the analyzed cytosolic substrate of bulk autophagy, was never hampered in the different test conditions. The reduced hydrolytic activity of the vacuoles in the *vps10*Δ strain seems to affect only large and complex substrates like organelles. It does not seem to affect the vacuolar breakdown of single cytosolic proteins like Pgk1 or protein/RNA-complexes like ribosomes. Because the remaining hydrolytic activity of the vacuole is sufficient for the efficient degradation of certain cytosolic proteins and complexes, it will be interesting to test whether overexpression or aggregation of these proteins may exceed the hydrolytic capacity of the vacuole.

The presented data and concept concerning the Vps10-dependent modulated targeting and maturation of Pep4 and the resulting selectively altered vacuolar activity have also an impact on other questions. The analysis and engineering of Pep4 targeting is of interest for different industrial applications^25,26^. The virulence of the plant pathogenic fungus *Ustilago maydis* is essentially depending on Pep4^27^. Moreover, Pep4 protects yeast cells against spontaneous prion generation^28^, while the loss of Pep4 activity results in a shortened lifespan^29,30^. Pep4 is closely related to mammalian aspartyl-proteases, such as the lysosomal cathepsin D^31^. Deficiencies in cathepsin D result in neurodegenerative disorders^32,33^. The mislocalization of pro-cathepsin D has been observed in several types of cancer cells^34^. Moreover, cathepsin D is tested as a molecular drug target in tumourigenesis^35,36^.

Therefore, the contribution of the vacuole/lysosome system and its regulation to cellular homeostasis is of great importance and essential for further understanding of protein aggregation disorders^8,37,38^.

Our data stress the importance to shift the analytical focus additionally to the targeting of cargos that is thought to rely on partially redundant factors. This might add further mosaic pieces to the analysis and understanding of complex disorders with Vps10-domain receptor contribution such as cancer^39^, cardiovascular and metabolic diseases^7^ or age-related neurodegenerative diseases like Alzheimer´s Disease^8,40^.

## Methods

### Yeast strains and culture conditions

The *Saccharomyces cerevisiae* strains and deletion mutants used in this study were purchased from EUROSCARF (Frankfurt, Germany). Yeast complete (YPD; 1% yeast extract, 2% peptone, 2% glucose, pH 7.4), selective minimal glucose media (SD; 0.3% glucose, 0.5% ammonium sulfate, 0.17% yeast nitrogen base without amino acids, auxotrophic amino acids and nucleoside, pH 6.0), synthetic glucose media (SD(+N); 2% glucose, 0.5% ammonium sulfate, 0.17% yeast nitrogen base without amino acids, auxotrophic amino acids and nucleoside, pH 6.0). Glycerol-containing medium was used for the proliferation of mitochondria (2% glycerol, 0.1% glucose, 0.5% ammonium sulfate, 0.17% yeast nitrogen base without amino acids, auxotrophic amino acids and nucleoside, pH 6.0) and oleate-containing medium for the proliferation of peroxisomes (0.5% ammonium sulfate, 0.17% yeast nitrogen base without amino acids, auxotrophic amino acids and nucleoside, 0.05% 20% Tween40, 0.1% oleic acid, pH 6.0) have been described previously^14^. Additional to oleate media, the oleate plates contained 0.1% yeast extract, 0.5% 20% Tween40 and 2.4% agar. The nitrogen-starvation medium (SD(-N)) contained 2% glucose, 0.17% yeast nitrogen base without amino acids, auxotrophic amino acids and nucleoside, adjusted to pH 6.0.

### Plasmids

In order to generate Pex11-GFP, 794 nucleotides upstream the PEX11 and the PEX11 open reading frame missing the stop codon were amplified by PCR with genomic *S. cerevisiae* (BY4742) DNA as a template and RE3850 (5′-AAAGAGCTCAAGAAGCTCAAATGAGCGGTT-3′) and RE3985 (5′-AAAGGATCCTGTAGCTTTCCACATGTCTTG-3′) as primers. The PCR product was digested with *Sac*I/*Bam*HI and cloned into pUG35, replacing the MET25 promotor.

The plasmid Pgk1-GFP was provided by Prof. Michael Thumm (Universität Göttingen, GER)^41^. The Pep4-GFP plasmid was a kind gift of Prof. David Goldfarb (University of Rochester, NY, USA)^42^. The DsRed-HDEL plasmid was a kind gift of Prof. Scott Emr (Cornell University, Ithaca, USA)^43^. The Tom70-GFP plasmid was a kind gift of Dr. Jan Schwichtenberg and Prof. Joachim Rassow (Ruhr-Universität Bochum, GER). Rpl25-GFP (94) was ordered from Prof. Michael Rout (Rockefeller University, New York City, NY, USA) via Addgene (Addgene plasmid #24037).

### Secretion assay

For the detection of secreted Pep4, yeast strains were grown exponentially in SD-medium, washed twice in water and then spotted as 10-fold serial dilutions directly on YPD plates as well as on nitrocellulose membranes placed on YPD plates. After incubation for 36 h at 30 °C, the nitrocellulose membrane were washed with wash-buffer and incubated with Pep4-antibodies.

### Pexophagy Assay

For the pexophagy assay based on^16^, yeast strains expressing the peroxisomal membrane protein Pex11 C-terminally fused with GFP were grown in two precultures (20 ml overnight and 50 ml for 8 h, OD_600nm_ = 0.3) in SD-medium at 30 °C. Peroxisomal proliferation was induced by incubating the cells for 16 h at 30 °C (OD_600nm_ = 0.5) in 100 ml oleate media. To induce pexophagy the cells first had to be harvested at 4000 rpm for 5 min at 4 °C and washed two times with 5 ml sterile dH_2_O (5 min, 4000 rpm, 4 °C). Cells were resuspended in 1 ml sterile water and 0.5 ml of cell suspension were transferred to 100 ml nitrogen-starvation-media (SD(-N)). Samples of the starting point (T0 samples, 0.5 ml remaining of cell suspension) were taken immediately, harvested for 5 min at 4000 rpm and prepared by TCA precipitation. The culture was incubated for 23 h at 30 °C. After 23 h the T23 samples (50 ml) were harvested, washed two times and as well prepared by TCA (trichloroacetic acid)-precipitation (as described in^44^).

### Bulk autophagy assay

To monitor bulk autophagy based on^16^, yeast cells expressing the cytosolic protein Pgk1 (3-phosphoglycerate kinase1) C-terminally fused with GFP were grown in three precultures (first preculture in 10 ml, second and third in 20 ml, OD_600nm_ = 0.1) under selective minimal glucose conditions at 30 °C. In order to initiate bulk autophagy, the third preculture (incubated for 16 h at 30 °C) were washed two times and resuspended in 1 ml sterile dH_2_O. 200 µl cell suspension were transferred to 20 ml SD-medium, which treated with finally 0.2 µg/ml rapamycin (Sigma-Aldrich) and/or DMSO as a control. As a starting point of bulk autophagy (T0) the remaining of cell suspension were immediately harvested at 4000 rpm for 5 min at 4 °C and prepared by TCA precipitation. After 23 h incubation at 30 °C the T23 samples were harvested (5 min, 4000 rpm at 4 °C), washed two times and then prepared by TCA precipitation (as described in^44^).

### Ribophagy assay

The ribophagy-assay were performed as previously described in^16^, with slight modifications. Yeast cells expressing Rpl25-GFP were incubated in 10 ml SD-medium for 8 h at 30 °C. The cells (OD_600nm_ = 0.1) were transferred to 20 ml SD(+N) medium, in which they were incubated for 16 h. Then, the cells (OD_600nm_ = 0.3) were transferred to 20 ml SD(+N) medium and were incubated for 8 h, followed by another culture (OD_600nm_ = 0.1) that was incubated for 12 h. After centrifugation, the cells were washed twice in water and resuspended in 1 ml sterile water. To obtain the T0 samples, 0.5 ml of cell suspension were TCA precipitated. To induce the degradation of ribosomes, 0.5 ml remaining of cell suspension were transferred in 20 ml SD(-N) medium and incubated for 6 h at 30 °C. Finally, the T6 samples were also TCA precipitated (as described in^44^).

### Mitophagy assay

Yeast cells expressing Tom70-GFP were grown in 10 ml SD-medium for 8 h in the first preculture. The cells (OD_600nm_ = 0.1) were transferred to 20 ml 2% glycerol/0.1% glucose medium, in which they were incubated for 12 h, followed by a third preculture (OD_600nm_ = 0.1) in 50 ml 2% glycerol/0.1% glucose medium for 12 h and a fourth preculture (OD_600nm_ = 0.1) in 100 ml 2% glycerol/0.1% glucose medium for 10 h. Finally, the cells were harvested and washed in water, before they were transferred to 100 ml SD(-N) medium (T0 samples). The cells were incubated for 23 h, before they were harvested and washed (T23 samples)^16^. The samples precipitated with TCA (as described in^44^).

### Immunodetection

Polyclonal rabbit antibodies were raised against Pep4 (a kind gift of Prof. Wolf, Stuttgart), Pex5^45^ and Por1^23^. Monoclonal mouse antibodies were raised against GFP (Roche). After elimination not bound primary antibody the blots were incubated with goat anti-mouse IRDye® 800CW or IRDye® 680RD goat anti-rabbit as secondary antibodies and visualized with the Odyssey® infrared imaging system (LI-COR Bioscience, Bad Homburg).

### Fluorescence microscopy

1.5 ml culture were pelleted at 4000 rpm for 5 min at room temperature and resuspended in 100 µl of FM4-64/YPD-medium (1/125 volume of 1 mM FM4-64, purchased from Invitrogen Karlsruhe (T3166). After cultivation for 30 min at 30 °C the cell culture was washed twice with YPD-medium and incubated in 1 ml fresh YPD-medium for 2 h at 30 °C using a rotator and an aluminium-foil to shield from the light. Before the cells were subjected to microscopy, they were harvested at 4000 rpm for 5 min at room temperature, washed twice with 1xPBS-buffer and then resuspended in 40 to 60 µl 1xPBS-buffer.

The analysis of live cells was performed with a Zeiss Axioplan microscope and deconvolved with AxioVision 4.1 software (Zeiss, Jena).

### Statistical analysis

The intensity of free GFP signals on the Western Blots was calculated by Image Studio Lite, LI-COR Bioscience (n = 5). The results are presented as means ± standard deviation (SD). The analysis of variance was performed by use of t-test procedures. A p-value p < 0.01(**) or p < 0.001(***) was considered as significant.

## Supplementary information

Supplementary Information

## Data Availability

The raw data generated during this study are available from the corresponding author on reasonable request.
