# Supplementary material for: Vps10-mediated targeting of Pep4 determines the activity of the vacuole in a substrate-dependent manner
Source: Sci Rep. 2019 Jul 22;9:10557. doi: 10.1038/s41598-019-47184-7 (PMC6646403; doi:10.1038/s41598-019-47184-7)
Supplement: Supplementary file 1 — Supplementary Information [file 41598_2019_47184_MOESM1_ESM.pdf]

Supplementary Information

Vps10-mediated targeting of Pep4 determines the activity of the vacuole in a substrate-dependent manner.

Fahd Boutouja[1], Christian M. Stiehm[1], Thomas Mastalski[1], Rebecca Brinkmeier[1], Christina Reidick[1], Fouzi El Magraoui[2] and Harald W. Platta[1,*]

[1]Biochemie Intrazellulärer Transportprozesse, Ruhr-Universität Bochum, 44801 Bochum, Germany
[2]Biomedizinische Forschung, Leibniz-Institute for Analytical Sciences (ISAS-e.V.), 44139 Dortmund,
Germany
*****corresponding: harald.platta@rub.de

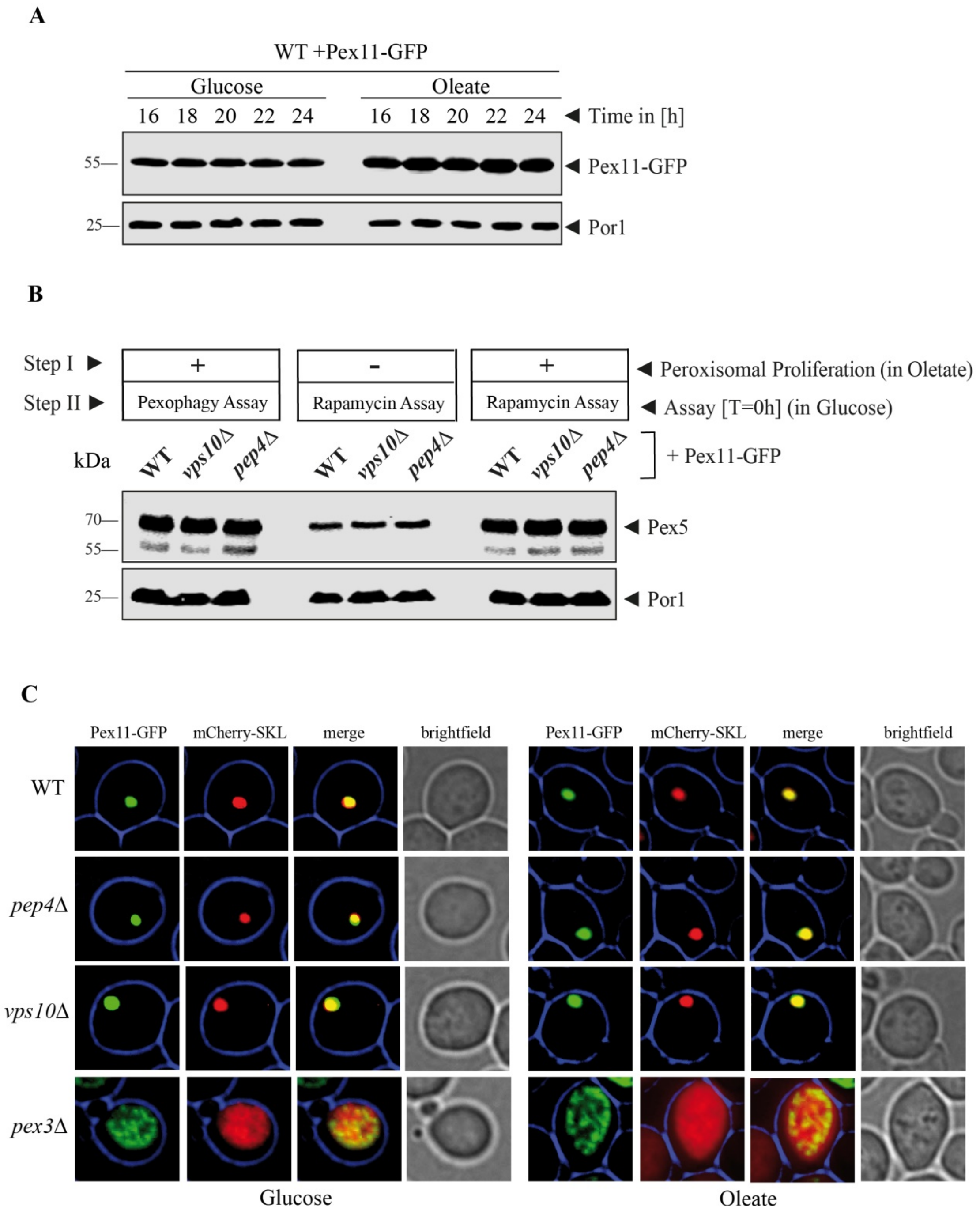

Supplementary Figure S1. Comparison of peroxisomal marker proteins in oleate-containing or glucose-based media. (A) Comparison of non-inducing (glucose) and peroxisomal proliferation-inducing (oleate) conditions. The protein level of Pex11-GFP is only slightly elevated, most likely because it is plasmid-encoded. (B) The protein level of the endogenous peroxin Pex5 is strongly elevated under peroxisomal proliferation-inducing conditions (oleate). Thus, more peroxins and therefore more peroxisomes are present at the beginning of the assays labeled with "+Proliferation" than without proliferation. (C) Pex11-GFP co-localizes with the peroxisomal matrix protein mCherry-SKL in WT, *pep4*Δ and *vps10*Δ cells and behaves like a peroxisomal protein and not as a cytosolic protein under glucose as well as under oleate conditions. The strain *pex3*Δ served as negative control. The full-length blots of Fig. S1A and S1B are presented in the Supplementary Fig. S4.

uncropped Figure 1a

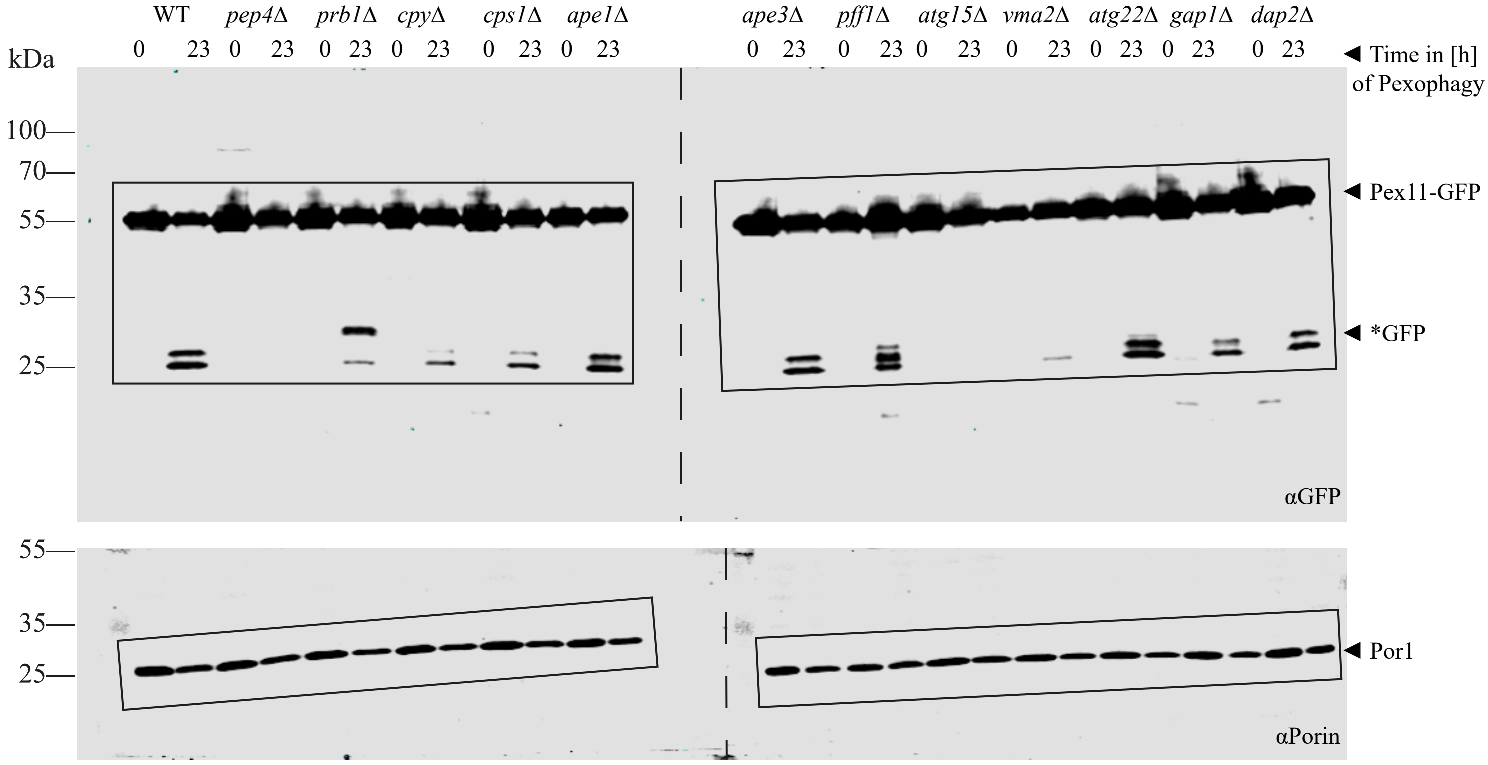

uncropped Figure 2a

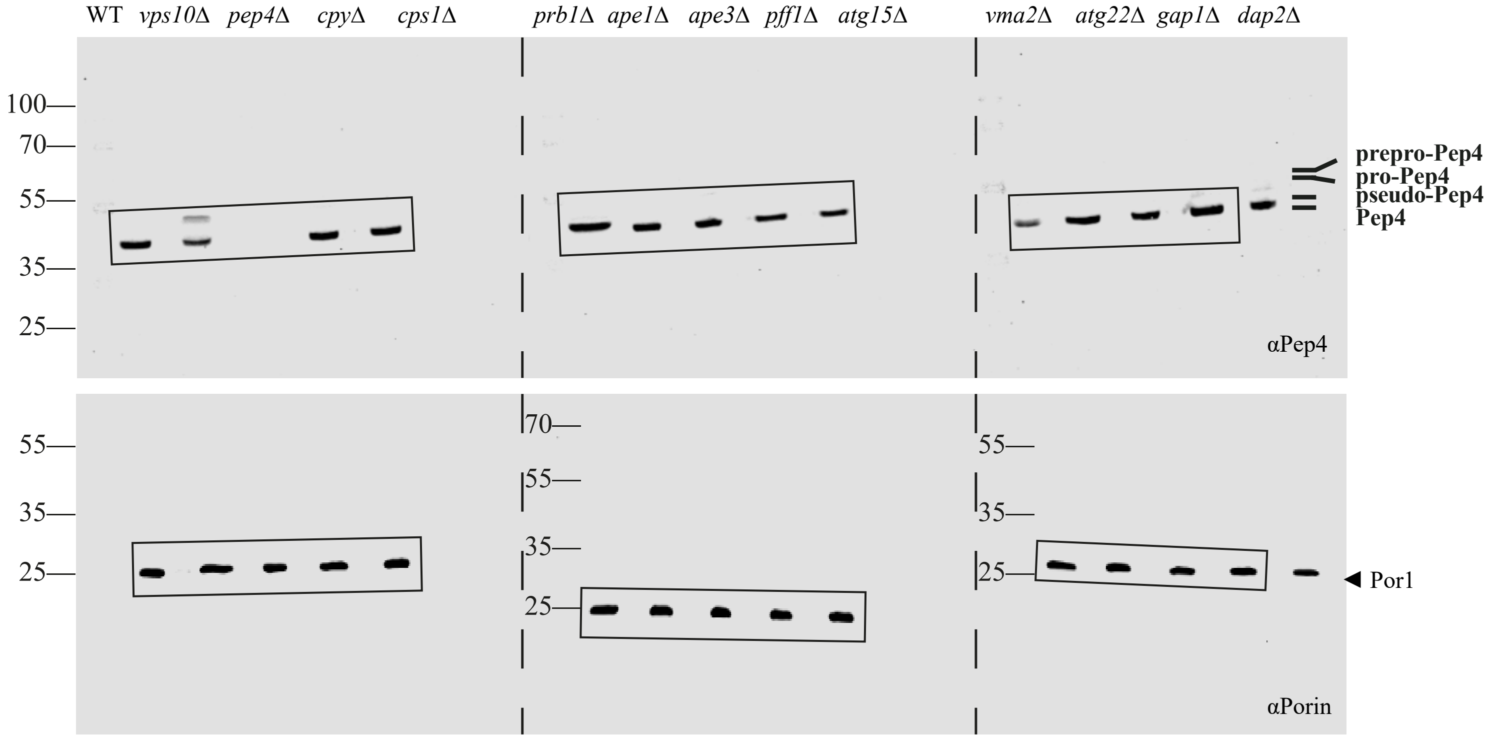

uncropped Figure 2d

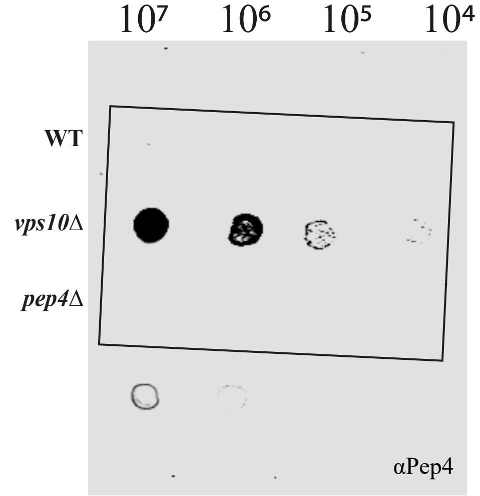

uncropped Figure 3b

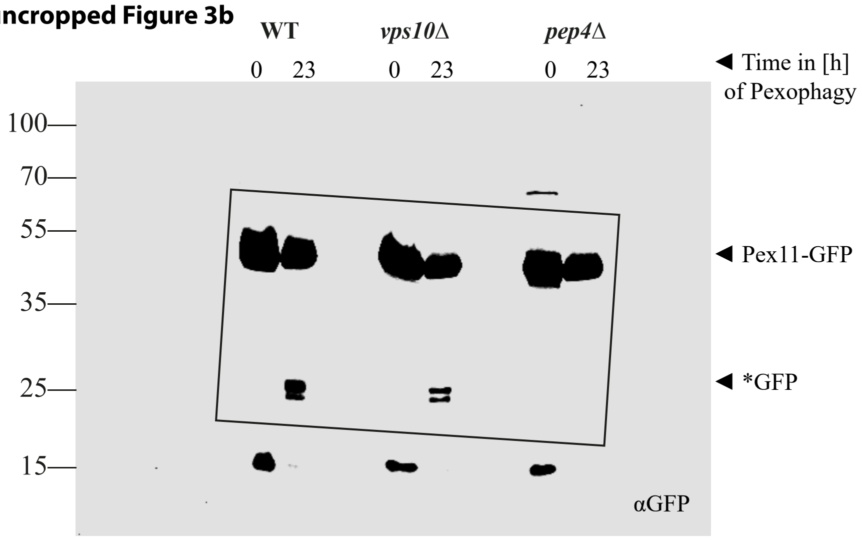

Supplementary Figure S2. Uncropped immunoblots. Boxes indicate the cropped panels.

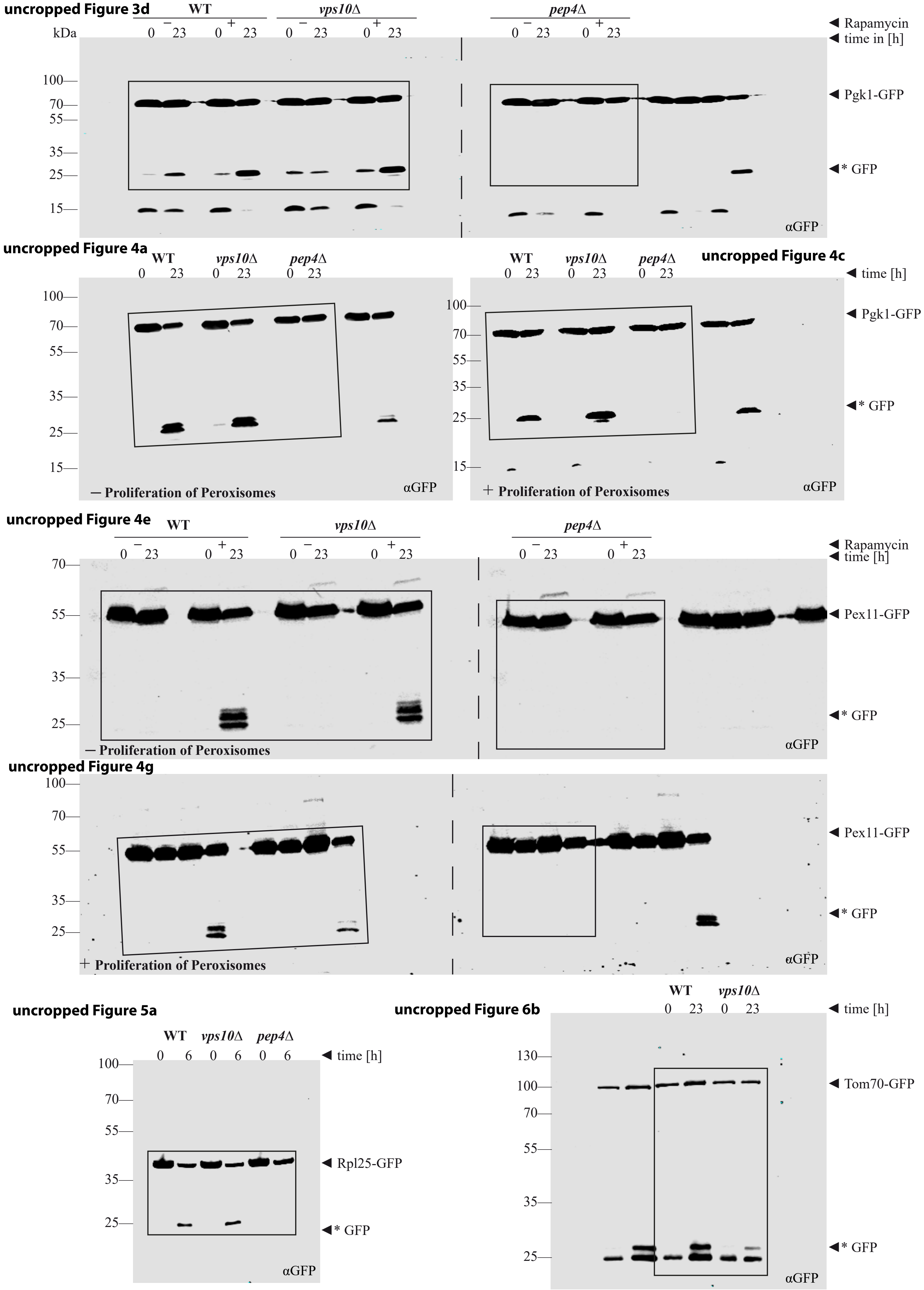

Supplementary Figure S3. Uncropped immunoblots. Boxes indicate the cropped panels.

uncropped Figure 2b

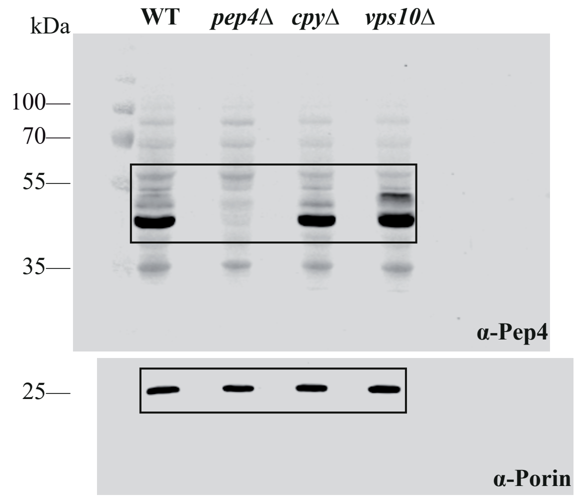

uncropped Figure S1a

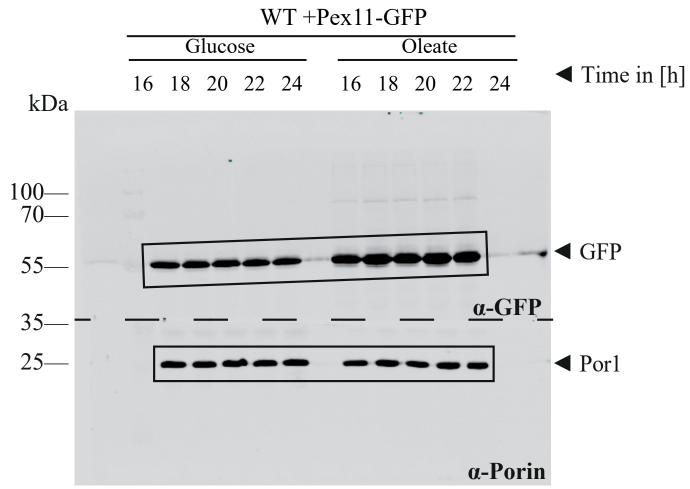

uncropped Figure S1b

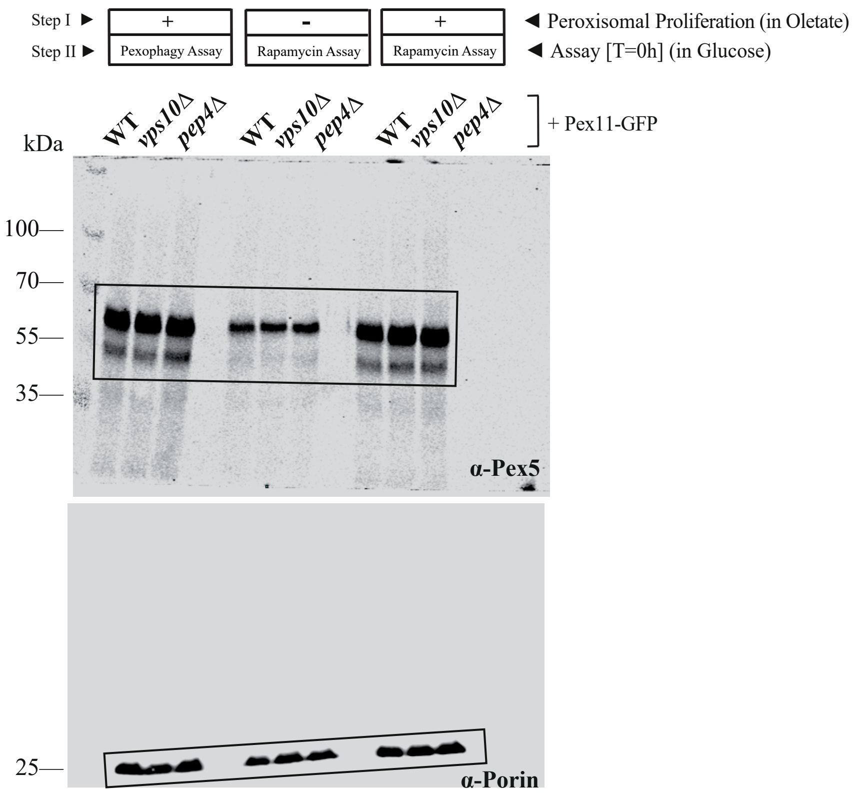

Supplementary Figure S4. Uncropped immunoblots. Boxes indicate the cropped panels.
